# (4*R*)-4-Benzyl-3-{(4*S*)-4-chloro-4-[(*S*)-2,2-dimethyl-1,3-dioxolan-4-yl]butano­yl}-1,3-oxazolidin-2-one

**DOI:** 10.1107/S1600536811053840

**Published:** 2011-12-17

**Authors:** Sandra Börding, Carsten Strohmann, Hans Preut, Martin Hiersemann

**Affiliations:** a Fakultät Chemie, Technische Universität Dortmund, Otto-Hahn-Strasse 6, 44221 Dortmund, Germany

## Abstract

The title compound, C_19_H_24_ClNO_5_, was synthesized and subsequently employed in an Evans alkyl­ation. The purpose was to prove the absolute configuration in the projected synthesis of the side chain of (–)-Lytophilippine A. The oxazolidinone and the isopropylidene acetal rings have twisted conformations. The oxazolidinone and side-chain carbonyl groups are orientated in an anti­periplanar arrangement to minimize van der Waals repulsions. Furthermore, the Cl atom and the acetonide-protected secondary alcohol are also in an anti­periplanar arrangement with a torsion angle of 173.64 (14)°. The absolute configuration was determined and agrees with the configuration of the used chiral auxiliary.

## Related literature

For background to the synthesis, see: Gille & Hiersemann (2010 ▶); Jang *et al.* (2011 ▶); Řezanka *et al.* (2004 ▶). For Evans alkyl­ation, see: Evans *et al.* (1981 ▶, 1982 ▶).
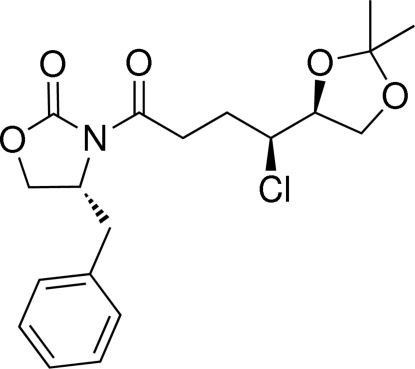


## Experimental

### 

#### Crystal data

C_19_H_24_ClNO_5_
*M* *_r_* = 381.84
Monoclinic, 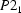

*a* = 11.7552 (9) Å
*b* = 5.9139 (4) Å
*c* = 13.8789 (11) Å
β = 109.023 (9)°
*V* = 912.16 (12) Å^3^
*Z* = 2
Mo *K*α radiation
μ = 0.24 mm^−1^
*T* = 173 K
0.40 × 0.20 × 0.20 mm

#### Data collection

Oxford Diffraction Xcalibur Sapphire3 diffractometer
Absorption correction: multi-scan (*CrysAlis RED*; Oxford Diffraction, 2008 ▶) *T*_min_ = 0.910, *T*_max_ = 0.954
6562 measured reflections
3594 independent reflections
2515 reflections with *I* > 2s(*I*)
*R*_int_ = 0.033

#### Refinement

*R*[*F* ^2^ > 2σ(*F* ^2^)] = 0.039
*wR*(*F* ^2^) = 0.047
*S* = 0.98
3594 reflections
237 parameters
1 restraint
H-atom parameters constrained
Δρ_max_ = 0.22 e Å^−3^
Δρ_min_ = −0.21 e Å^−3^
Absolute structure: Flack (1983 ▶), 1198 Friedel pairs
Flack parameter: 0.11 (5)

### 

Data collection: *CrysAlis CCD* (Oxford Diffraction, 2008 ▶); cell refinement: *CrysAlis CCD*; data reduction: *CrysAlis RED* (Oxford Diffraction, 2008 ▶); program(s) used to solve structure: *SHELXS97* (Sheldrick, 2008 ▶); program(s) used to refine structure: *SHELXL97* (Sheldrick, 2008 ▶); molecular graphics: *SHELXTL-Plus* (Sheldrick, 2008 ▶) and *PLATON* (Spek, 2009 ▶); software used to prepare material for publication: *SHELXTL-Plus*.

## Supplementary Material

Crystal structure: contains datablock(s) I, global. DOI: 10.1107/S1600536811053840/ff2045sup1.cif

Structure factors: contains datablock(s) I. DOI: 10.1107/S1600536811053840/ff2045Isup2.hkl

Supplementary material file. DOI: 10.1107/S1600536811053840/ff2045Isup3.cml

Additional supplementary materials:  crystallographic information; 3D view; checkCIF report

## Figures and Tables

**Fig. 1.**
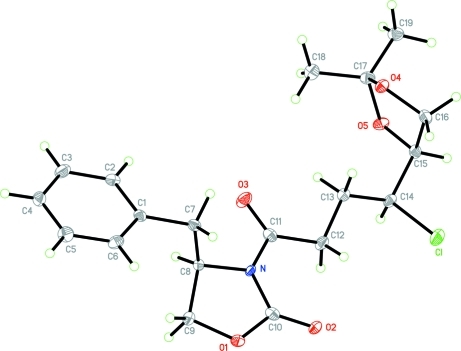
The molecular structure of the title compound, showing the labelling of all non-H atoms. Displacement ellipsoids are shown at the 30% probability level.

## supplementary crystallographic information

### Comment

The title compound I was obtained during the synthesis of the side chain of (–)-Lytophilippine A (Řezanka *et al.*, 2004). Recently our research group published the synthesis of the core fragment of (–)-Lytophilippine A (Gille & Hiersemann, 2010). Shortly after, the first total synthesis of the postulated structure was published (Jang *et al.*, 2011). Compound I was synthesized and subsequently applied in an Evans alkylation (Evans *et al.*, 1981; Evans *et al.*, 1982) to install the stereogenic center at C23. The synthesis of I was carried out from carboxylic acid and (*R*)-4-benzyloxazolidin-2-one.

The oxazolidinone ring adopts a nearly coplanar conformation and the isopropylidene acetal is an open envelope-like structure. The oxazolidinone- and side-chain carbonyl groups are orientated in an antiperiplanar arrangement to minimize van der Waals repulsions. The dihedral angle between the plane through N, C10, O2 and the plane through N, C11, O3 is 9.4 (4)°. Furthermore, the chlorine atom and the acetonide protected secondary alcohol are also in an antiperiplanar arrangement with an torsion angle of 173.64 (14)°. The absolute configuration was determined and agrees with the configuration of the used chiral auxiliary.

### Experimental

To a solution of carboxylic acid (840 mg, 3.77 mmol, 1.0 eq) in THF (20 ml, 5 ml/mmol) was added Et_3_N (1.14 ml, 8.15 mmol, 2.0 eq) and pivaloylchloride (0.6 ml, 4.89 mmol, 1.2 eq) at 253 K. After stirring at this temperature for 2 h the mixture was warmed to 273 K. Solid LiCl (240 mg, 5.67 mmol, 1.5 eq) and (*R*)-4-benzyloxazolidin-2-one (669 mg, 3.77 mmol, 1.0 eq) were added. The reaction was quenched with H_2_O after stirring for 2 h at room temperature. The layers were separated and the aqueous phase extracted with Et_2_O. The combined organic phases were dried over anhydrous MgSO_4_, filtered and concentrated under reduced pressure. Flash column chromatography (cyclohexane/ethyl acetate 5/1) afforded the title compound (1.12 g, 2.93 mmol, 84%) as a thick colourless oil. Single crystals of (I) were obtained by crystallization from isohexane to provide white needles. *R_f_* 0.43 (cyclohexane/ethyl acetate 2/1); Anal. Calcd. for C_19_H_24_ClNO_5_: C,59.8; H, 6.3; N, 3.7; Found: C, 59.8; H, 6.5; N, 3.6; [α]_D_^20^ -51.4 (c 1.02, CH_3_Cl); *M* = 381.85 g/mol.

### Refinement

The hydrogen atoms were placed in calculated posions with C–H bond distances in the range from 0.95 to 1.00 Å and refined as riding on their parent atoms with *U*_iso_ = 1.2 or 1.5 *x U*_eq_(C).

### Crystal data

**Table:**

C_19_H_24_ClNO_5_	*F*(000) = 404
*M**_r_* = 381.84	*D*_x_ = 1.390 Mg m^−^^3^
Monoclinic, *P*2_1_	Mo *K*α radiation, λ = 0.71073 Å
Hall symbol: P 2yb	Cell parameters from 2793 reflections
*a* = 11.7552 (9) Å	θ = 2.8–29.0°
*b* = 5.9139 (4) Å	µ = 0.24 mm^−^^1^
*c* = 13.8789 (11) Å	*T* = 173 K
β = 109.023 (9)°	Block, white
*V* = 912.16 (12) Å^3^	0.40 × 0.20 × 0.20 mm
*Z* = 2	

### Data collection

**Table:**

Oxford Diffraction Xcalibur Sapphire3 diffractometer	3594 independent reflections
Radiation source: Enhance (Mo) X-ray Source	2515 reflections with *I* > 2s(*I*)
graphite	*R*_int_ = 0.033
Detector resolution: 16.0560 pixels mm^-1^	θ_max_ = 26.0°, θ_min_ = 2.8°
ω scans	*h* = −14→14
Absorption correction: multi-scan (*CrysAlis RED*; Oxford Diffraction, 2008)	*k* = −7→7
*T*_min_ = 0.910, *T*_max_ = 0.954	*l* = −17→16
6562 measured reflections	

### Refinement

**Table:**

Refinement on *F*^2^	Secondary atom site location: difference Fourier map
Least-squares matrix: full	Hydrogen site location: inferred from neighbouring sites
*R*[*F*^2^ > 2σ(*F*^2^)] = 0.039	H-atom parameters constrained
*wR*(*F*^2^) = 0.047	*w* = 1/[σ^2^(*F*_o_^2^) + (0.007*P*)^2^] where *P* = (*F*_o_^2^ + 2*F*_c_^2^)/3
*S* = 0.98	(Δ/σ)_max_ < 0.001
3594 reflections	Δρ_max_ = 0.22 e Å^−^^3^
237 parameters	Δρ_min_ = −0.21 e Å^−^^3^
1 restraint	Absolute structure: Flack (1983), 1198 Friedel pairs
Primary atom site location: structure-invariant direct methods	Flack parameter: 0.11 (5)

### Special details

**Table:**

Experimental. CrysAlis RED (Oxford Diffraction, 2008), Empirical absorption correction using spherical harmonics, implemented in SCALE3 ABSPACK scaling algorithm.
Geometry. All e.s.d.'s (except the e.s.d. in the dihedral angle between two l.s. planes) are estimated using the full covariance matrix. The cell e.s.d.'s are taken into account individually in the estimation of e.s.d.'s in distances, angles and torsion angles; correlations between e.s.d.'s in cell parameters are only used when they are defined by crystal symmetry. An approximate (isotropic) treatment of cell e.s.d.'s is used for estimating e.s.d.'s involving l.s. planes.
Refinement. Refinement of *F*^2^ against ALL reflections. The weighted *R*-factor *wR* and goodness of fit *S* are based on *F*^2^, conventional *R*-factors *R* are based on *F*, with *F* set to zero for negative *F*^2^. The threshold expression of *F*^2^ > σ(*F*^2^) is used only for calculating *R*-factors(gt) *etc*. and is not relevant to the choice of reflections for refinement. *R*-factors based on *F*^2^ are statistically about twice as large as those based on *F*, and *R*- factors based on ALL data will be even larger.

### Fractional atomic coordinates and isotropic or equivalent isotropic displacement parameters (Å^2^)

**Table:**

	*x*	*y*	*z*	*U*_iso_*/*U*_eq_	
C1	0.1657 (2)	−0.3801 (4)	0.17220 (18)	0.0189 (6)	
C2	0.0920 (2)	−0.2107 (4)	0.18929 (18)	0.0224 (7)	
H2	0.1267	−0.0727	0.2202	0.027*	
C3	−0.0317 (2)	−0.2418 (4)	0.1616 (2)	0.0290 (7)	
H3	−0.0810	−0.1269	0.1750	0.035*	
C4	−0.0827 (2)	−0.4398 (4)	0.11451 (19)	0.0270 (7)	
H4	−0.1675	−0.4598	0.0940	0.032*	
C5	−0.0110 (2)	−0.6085 (4)	0.0972 (2)	0.0285 (7)	
H5	−0.0459	−0.7454	0.0653	0.034*	
C6	0.1125 (2)	−0.5773 (4)	0.12673 (19)	0.0245 (7)	
H6	0.1616	−0.6951	0.1153	0.029*	
C7	0.30014 (18)	−0.3420 (4)	0.20129 (17)	0.0201 (6)	
H7A	0.3425	−0.4887	0.2185	0.024*	
H7B	0.3273	−0.2439	0.2623	0.024*	
C8	0.3321 (2)	−0.2315 (4)	0.11426 (18)	0.0191 (6)	
H8	0.2796	−0.0967	0.0885	0.023*	
C9	0.3254 (2)	−0.3926 (4)	0.02582 (19)	0.0312 (7)	
H9A	0.2762	−0.5272	0.0279	0.037*	
H9B	0.2896	−0.3155	−0.0405	0.037*	
C10	0.5254 (2)	−0.3120 (5)	0.10476 (17)	0.0206 (6)	
C11	0.4985 (2)	0.0280 (4)	0.20304 (19)	0.0216 (6)	
C12	0.6276 (2)	0.0996 (4)	0.2280 (2)	0.0244 (7)	
H12A	0.6385	0.1773	0.1684	0.029*	
H12B	0.6800	−0.0358	0.2430	0.029*	
C13	0.6643 (2)	0.2563 (4)	0.31873 (19)	0.0220 (7)	
H13A	0.6713	0.1670	0.3808	0.026*	
H13B	0.5995	0.3689	0.3106	0.026*	
C14	0.7817 (2)	0.3812 (4)	0.33466 (19)	0.0209 (6)	
H14	0.7708	0.4861	0.2758	0.025*	
C15	0.8252 (2)	0.5187 (4)	0.43295 (18)	0.0209 (6)	
H15	0.9058	0.5866	0.4413	0.025*	
C16	0.8292 (2)	0.3846 (4)	0.52813 (18)	0.0244 (6)	
H16A	0.8405	0.2212	0.5187	0.029*	
H16B	0.8949	0.4389	0.5886	0.029*	
C17	0.6855 (2)	0.6563 (4)	0.50682 (18)	0.0229 (6)	
C18	0.55163 (18)	0.6806 (5)	0.46026 (17)	0.0278 (6)	
H18A	0.5204	0.5595	0.4104	0.042*	
H18B	0.5328	0.8275	0.4261	0.042*	
H18C	0.5143	0.6706	0.5138	0.042*	
C19	0.7398 (2)	0.8166 (4)	0.59485 (18)	0.0272 (7)	
H19A	0.7155	0.7708	0.6531	0.041*	
H19B	0.7114	0.9706	0.5743	0.041*	
H19C	0.8277	0.8121	0.6141	0.041*	
Cl	0.90160 (5)	0.18577 (11)	0.33911 (5)	0.03519 (19)	
N	0.46002 (17)	−0.1656 (3)	0.14456 (15)	0.0161 (5)	
O1	0.44892 (15)	−0.4571 (3)	0.04026 (13)	0.0265 (4)	
O2	0.63229 (13)	−0.3207 (3)	0.12130 (12)	0.0242 (4)	
O3	0.42567 (14)	0.1337 (3)	0.22968 (13)	0.0314 (5)	
O4	0.71441 (14)	0.4278 (2)	0.53829 (13)	0.0204 (4)	
O5	0.73891 (12)	0.6923 (3)	0.42756 (11)	0.0231 (4)	

### Atomic displacement parameters (Å^2^)

**Table:**

	*U*^11^	*U*^22^	*U*^33^	*U*^12^	*U*^13^	*U*^23^
C1	0.0209 (14)	0.0224 (16)	0.0130 (15)	−0.0011 (12)	0.0049 (13)	0.0024 (11)
C2	0.0283 (16)	0.0211 (14)	0.0183 (17)	0.0020 (13)	0.0085 (15)	−0.0011 (12)
C3	0.0288 (17)	0.0318 (17)	0.0305 (19)	0.0072 (14)	0.0151 (16)	−0.0004 (14)
C4	0.0206 (16)	0.0364 (16)	0.0244 (18)	−0.0029 (14)	0.0079 (15)	0.0005 (14)
C5	0.0306 (17)	0.0216 (16)	0.0353 (19)	−0.0098 (14)	0.0132 (16)	−0.0060 (14)
C6	0.0290 (17)	0.0195 (14)	0.0296 (18)	0.0021 (13)	0.0158 (15)	0.0014 (13)
C7	0.0225 (13)	0.0186 (14)	0.0183 (15)	−0.0005 (14)	0.0054 (12)	−0.0032 (12)
C8	0.0156 (14)	0.0228 (14)	0.0176 (16)	0.0021 (11)	0.0038 (13)	−0.0029 (12)
C9	0.0176 (14)	0.0477 (19)	0.0294 (18)	−0.0047 (14)	0.0094 (15)	−0.0112 (14)
C10	0.0278 (14)	0.0202 (13)	0.0131 (14)	−0.0007 (16)	0.0058 (13)	0.0073 (14)
C11	0.0259 (17)	0.0204 (14)	0.0168 (16)	−0.0011 (13)	0.0046 (14)	0.0036 (13)
C12	0.0197 (14)	0.0233 (15)	0.0334 (18)	−0.0028 (12)	0.0132 (14)	−0.0045 (12)
C13	0.0198 (14)	0.0239 (16)	0.0230 (17)	0.0007 (12)	0.0078 (14)	−0.0014 (12)
C14	0.0198 (15)	0.0208 (14)	0.0239 (17)	0.0079 (13)	0.0097 (14)	0.0060 (13)
C15	0.0176 (15)	0.0202 (14)	0.0242 (17)	−0.0038 (12)	0.0058 (14)	−0.0033 (13)
C16	0.0222 (16)	0.0280 (15)	0.0205 (17)	−0.0031 (13)	0.0033 (14)	−0.0005 (13)
C17	0.0277 (14)	0.0224 (15)	0.0215 (16)	−0.0027 (15)	0.0122 (14)	−0.0001 (14)
C18	0.0277 (14)	0.0233 (13)	0.0337 (17)	−0.0015 (16)	0.0116 (14)	0.0011 (15)
C19	0.0309 (17)	0.0260 (15)	0.0279 (18)	−0.0057 (12)	0.0140 (16)	−0.0055 (13)
Cl	0.0234 (4)	0.0399 (4)	0.0430 (5)	0.0074 (4)	0.0119 (4)	−0.0058 (4)
N	0.0116 (12)	0.0175 (12)	0.0205 (14)	0.0006 (9)	0.0070 (11)	−0.0043 (9)
O1	0.0241 (11)	0.0301 (10)	0.0254 (12)	−0.0011 (8)	0.0083 (10)	−0.0127 (9)
O2	0.0193 (8)	0.0274 (9)	0.0273 (11)	0.0067 (10)	0.0096 (8)	−0.0029 (10)
O3	0.0252 (9)	0.0262 (11)	0.0473 (14)	−0.0023 (9)	0.0182 (10)	−0.0133 (9)
O4	0.0215 (11)	0.0197 (9)	0.0234 (11)	0.0000 (9)	0.0120 (9)	0.0040 (8)
O5	0.0296 (10)	0.0209 (9)	0.0241 (10)	0.0017 (10)	0.0158 (9)	0.0022 (10)

### Geometric parameters (Å, °)

**Table:**

C1—C6	1.376 (3)	C11—C12	1.503 (3)
C1—C2	1.394 (3)	C12—C13	1.508 (3)
C1—C7	1.515 (3)	C12—H12A	0.9900
C2—C3	1.390 (3)	C12—H12B	0.9900
C2—H2	0.9500	C13—C14	1.517 (3)
C3—C4	1.379 (3)	C13—H13A	0.9900
C3—H3	0.9500	C13—H13B	0.9900
C4—C5	1.377 (3)	C14—C15	1.526 (3)
C4—H4	0.9500	C14—Cl	1.808 (2)
C5—C6	1.386 (3)	C14—H14	1.0000
C5—H5	0.9500	C15—O5	1.428 (2)
C6—H6	0.9500	C15—C16	1.528 (3)
C7—C8	1.524 (3)	C15—H15	1.0000
C7—H7A	0.9900	C16—O4	1.425 (3)
C7—H7B	0.9900	C16—H16A	0.9900
C8—N	1.476 (3)	C16—H16B	0.9900
C8—C9	1.536 (3)	C17—O4	1.426 (3)
C8—H8	1.0000	C17—O5	1.449 (2)
C9—O1	1.450 (2)	C17—C18	1.500 (3)
C9—H9A	0.9900	C17—C19	1.514 (3)
C9—H9B	0.9900	C18—H18A	0.9800
C10—O2	1.202 (2)	C18—H18B	0.9800
C10—O1	1.350 (3)	C18—H18C	0.9800
C10—N	1.386 (3)	C19—H19A	0.9800
C11—O3	1.211 (2)	C19—H19B	0.9800
C11—N	1.391 (3)	C19—H19C	0.9800
			
C6—C1—C2	118.2 (2)	C12—C13—C14	114.81 (19)
C6—C1—C7	121.8 (2)	C12—C13—H13A	108.6
C2—C1—C7	120.0 (2)	C14—C13—H13A	108.6
C3—C2—C1	120.7 (2)	C12—C13—H13B	108.6
C3—C2—H2	119.7	C14—C13—H13B	108.6
C1—C2—H2	119.7	H13A—C13—H13B	107.5
C4—C3—C2	119.8 (2)	C13—C14—C15	114.5 (2)
C4—C3—H3	120.1	C13—C14—Cl	110.85 (15)
C2—C3—H3	120.1	C15—C14—Cl	106.22 (17)
C5—C4—C3	120.1 (3)	C13—C14—H14	108.4
C5—C4—H4	120.0	C15—C14—H14	108.4
C3—C4—H4	120.0	Cl—C14—H14	108.4
C4—C5—C6	119.6 (2)	O5—C15—C14	108.09 (19)
C4—C5—H5	120.2	O5—C15—C16	103.84 (17)
C6—C5—H5	120.2	C14—C15—C16	113.78 (18)
C1—C6—C5	121.6 (2)	O5—C15—H15	110.3
C1—C6—H6	119.2	C14—C15—H15	110.3
C5—C6—H6	119.2	C16—C15—H15	110.3
C1—C7—C8	110.94 (19)	O4—C16—C15	103.26 (18)
C1—C7—H7A	109.5	O4—C16—H16A	111.1
C8—C7—H7A	109.5	C15—C16—H16A	111.1
C1—C7—H7B	109.5	O4—C16—H16B	111.1
C8—C7—H7B	109.5	C15—C16—H16B	111.1
H7A—C7—H7B	108.0	H16A—C16—H16B	109.1
N—C8—C7	112.25 (19)	O4—C17—O5	104.61 (18)
N—C8—C9	99.97 (18)	O4—C17—C18	109.5 (2)
C7—C8—C9	113.96 (18)	O5—C17—C18	108.19 (19)
N—C8—H8	110.1	O4—C17—C19	110.5 (2)
C7—C8—H8	110.1	O5—C17—C19	110.32 (19)
C9—C8—H8	110.1	C18—C17—C19	113.3 (2)
O1—C9—C8	105.4 (2)	C17—C18—H18A	109.5
O1—C9—H9A	110.7	C17—C18—H18B	109.5
C8—C9—H9A	110.7	H18A—C18—H18B	109.5
O1—C9—H9B	110.7	C17—C18—H18C	109.5
C8—C9—H9B	110.7	H18A—C18—H18C	109.5
H9A—C9—H9B	108.8	H18B—C18—H18C	109.5
O2—C10—O1	121.9 (3)	C17—C19—H19A	109.5
O2—C10—N	129.2 (3)	C17—C19—H19B	109.5
O1—C10—N	108.97 (19)	H19A—C19—H19B	109.5
O3—C11—N	118.3 (2)	C17—C19—H19C	109.5
O3—C11—C12	123.1 (2)	H19A—C19—H19C	109.5
N—C11—C12	118.6 (2)	H19B—C19—H19C	109.5
C11—C12—C13	110.96 (19)	C10—N—C11	129.0 (2)
C11—C12—H12A	109.4	C10—N—C8	111.57 (19)
C13—C12—H12A	109.4	C11—N—C8	119.42 (19)
C11—C12—H12B	109.4	C10—O1—C9	110.18 (18)
C13—C12—H12B	109.4	C16—O4—C17	106.18 (16)
H12A—C12—H12B	108.0	C15—O5—C17	109.33 (17)
			
C6—C1—C2—C3	0.4 (4)	O2—C10—N—C11	9.7 (4)
C7—C1—C2—C3	179.0 (2)	O1—C10—N—C11	−170.7 (2)
C1—C2—C3—C4	−1.5 (4)	O2—C10—N—C8	−172.6 (3)
C2—C3—C4—C5	1.5 (4)	O1—C10—N—C8	6.9 (3)
C3—C4—C5—C6	−0.4 (4)	O3—C11—N—C10	179.5 (2)
C2—C1—C6—C5	0.7 (4)	C12—C11—N—C10	0.9 (3)
C7—C1—C6—C5	−177.9 (2)	O3—C11—N—C8	2.0 (3)
C4—C5—C6—C1	−0.7 (4)	C12—C11—N—C8	−176.5 (2)
C6—C1—C7—C8	90.3 (3)	C7—C8—N—C10	104.9 (2)
C2—C1—C7—C8	−88.2 (3)	C9—C8—N—C10	−16.3 (2)
C1—C7—C8—N	172.7 (2)	C7—C8—N—C11	−77.2 (3)
C1—C7—C8—C9	−74.6 (3)	C9—C8—N—C11	161.60 (19)
N—C8—C9—O1	19.2 (2)	O2—C10—O1—C9	−173.6 (2)
C7—C8—C9—O1	−100.7 (2)	N—C10—O1—C9	6.8 (3)
O3—C11—C12—C13	21.4 (3)	C8—C9—O1—C10	−17.1 (2)
N—C11—C12—C13	−160.2 (2)	C15—C16—O4—C17	−35.9 (2)
C11—C12—C13—C14	−166.14 (19)	O5—C17—O4—C16	33.2 (2)
C12—C13—C14—C15	−173.5 (2)	C18—C17—O4—C16	148.95 (19)
C12—C13—C14—Cl	−53.4 (2)	C19—C17—O4—C16	−85.6 (2)
C13—C14—C15—O5	−63.7 (2)	C14—C15—O5—C17	116.1 (2)
Cl—C14—C15—O5	173.64 (14)	C16—C15—O5—C17	−5.0 (2)
C13—C14—C15—C16	51.1 (3)	O4—C17—O5—C15	−16.6 (2)
Cl—C14—C15—C16	−71.6 (2)	C18—C17—O5—C15	−133.4 (2)
O5—C15—C16—O4	24.8 (2)	C19—C17—O5—C15	102.2 (2)
C14—C15—C16—O4	−92.5 (2)		
